# Supplementary material for: Smooth Interpolating Curves with Local Control and Monotone Alternating Curvature
Source: Comput Graph Forum. 2022 Oct 6;41(5):25–38. doi: 10.1111/cgf.14600 (PMC9827861; doi:10.1111/cgf.14600)
Supplement: Supplementary file 1 — Supplement Material [file CGF-41-25-s001.zip › Local-Smooth-Interpolating-MonoCurvature/extern/clothoids/submodules/Utils/ThirdParties/fmt/fmt-8.0.1.zip!/fmt-8.0.1/support/rtd/theme/layout.html]

{% extends "basic/layout.html" %}
{% block extrahead %}

Page Redirection
{% endblock %}
{% block document %}
If you are not redirected automatically, follow the link to the fmt documentation.
{% endblock %}
{% block footer %}
{% endblock %}
